# Regional Differences in PM_2.5_ Chemical Composition and Inhalation Risk Assessment: A Case Study of Seoul, Incheon, and Wonju

**DOI:** 10.3390/toxics13040240

**Published:** 2025-03-24

**Authors:** Seung-Hyun Jung, Seon-Ho Baek, Shin-Young Park, Cheol-Min Lee, Jung-Il Lee

**Affiliations:** 1 Climate Technology Center, Korea Testing & Research Institute, Gwacheon 13810, Republic of Korea; ggi1118@ktr.or.kr; 2 Air Quality Center, Korea Testing & Research Institute, Gwacheon 13810, Republic of Korea; seono@ktr.or.kr; 3 Department of Environmental & Chemical Engineering, Seokyeong University, Seoul 02713, Republic of Korea; tlsdud060900@skuniv.ac.kr (S.-Y.P.); cheolmin@skuniv.ac.kr (C.-M.L.)

**Keywords:** PM_2.5_, region-specific, health risk assessment, Cr(IV), heavy metal

## Abstract

This study evaluates the chemical components of an aerodynamic diameter less than 2.5 μm (PM_2.5_) and its health risks in Seoul, Incheon, and Wonju, South Korea. The results revealed significant regional variations, particularly under the reasonable maximum exposure scenario, with Seoul’s average daily dose (6.4 × 10^−1^ µg/kg/day) approximately 2 times higher than Incheon (5.8 × 10^−1^ µg/kg/day) and Wonju (3.2 × 10^−1^ µg/kg/day) under the central tendency exposure scenario. Furthermore, exposure to the chemical components comprising PM_2.5_ can surpass risk thresholds when PM_2.5_ concentrations exceed the national standard levels. These findings suggest the potential benefits of preventive measures, such as minimizing outdoor exposure, especially for individuals over 60 years of age, to help reduce health risks. However, further research is needed to confirm the effectiveness of these measures in different regions. The study also highlighted the variation in the health impacts of PM_2.5_ concentrations and its chemical components across the different regions. The results suggest that relying solely on PM_2.5_ concentrations for health risk assessments may underestimate the risks associated with carcinogenic components such as chromium (Cr, VI). However, under the reasonable maximum exposure (RME) scenario, the excess cancer risk (ECR) for Cr(VI) exceeds the acceptable threshold in all three regions, suggesting a high carcinogenic risk under the RME scenario. For example, the ECR for Cr(VI) in Seoul was calculated as 1.4 × 10^−4^, Incheon as 2.0 × 10^−4^, and Wonju as 1.2 × 10^−4^. Therefore, we emphasize the importance of incorporating both the mass concentration of PM_2.5_ and its chemical constituents when conducting health risk assessments to inform region-specific health policies to mitigate health risks, particularly for vulnerable populations.

## 1. Introduction

Among the environmental factors affecting the global disease burden, air pollution has been evaluated as the most significant contributor to mortality, exhibiting a greater impact than other major risk factors, such as low physical activity, excessive sodium intake, and a high-cholesterol diet [1,2,3]. Air pollutants are complex mixtures comprising particulate matter (PM), gaseous contaminants, and heavy metals. Approximately 99% of the global population is exposed to air pollution levels that exceed the WHO air quality standards, rendering it a significant public health concern worldwide [4].

Among air pollutants, PM_2.5_, defined as particulate matter with an aerodynamic diameter of 2.5 μm or less, has been consistently reported to have adverse effects on human health [5,6]. The health impacts of PM_2.5_ exposure have been assessed in several studies [6,7], with PM_2.5_ mass concentration long being used as a benchmark for exposure [8,9]. However, due to its large surface area, PM_2.5_ can adsorb harmful chemicals and has a complex chemical composition [10]. The major components of PM_2.5_ include ions such as NO_3_^−^, SO_4_^2−^, Na^+^, and Cl^−^; carbonaceous components like elemental carbon and organic carbon; and trace elements such as Al, Ti, Cr, Zn, and Mn [11,12]. Heavy metals such as Cr, Zn, As, and Pb have been shown to have harmful effects, including carcinogenicity, even at low concentrations [13,14], and are associated with diseases such as asthma, pneumonia, lung cancer, and stroke [15]. In particular, heavy metals adsorbed onto PM_2.5_ can act as risk factors for lung cancer [16]. The main routes of exposure to heavy metals in PM_2.5_ include inhalation, ingestion, and dermal contact [17,18], and they are particularly known to have negative health effects during respiratory exposure [19]. Therefore, quantifying the chemical composition of PM_2.5_ is crucial for an accurate health impact assessment.

The content of PM_2.5_ chemical components varies by emission sources, leading to geographical differences in health impacts [7,20,21]. As a result, numerous studies have focused on understanding the health risks posed by the chemicals adsorbed onto PM_2.5_ [15,22]. For example, Li et al. [23] detected Pb, Al, and Cu in the blood and organs of rats exposed to PM_2.5_, confirming the potential accumulation of heavy metals in body tissues. Therefore, to evaluate the health effects of PM_2.5_ exposure, it is necessary to consider not only its concentration but also the toxicity and exposure levels of its chemical components.

This study was part of the national research project “Core Technology Development for Prevention and Management of Environmental Diseases” funded by the Ministry of Environment. The project aims to develop core technologies to investigate and prevent the impacts of PM_2.5_ and its adsorbed chemical components, focusing on neurological diseases such as stroke, dementia, and Parkinson’s disease. To achieve this, a cohort study was conducted with individuals aged 60 and older from three regions with different atmospheric conditions: Seoul, Incheon, and Wonju. The study aimed to assess the effects of PM_2.5_ exposure and its chemical components on neurological disease incidence and to develop prevention and management technologies. In particular, the study emphasized the importance of assessing both PM_2.5_ concentration and the types and exposure levels of its chemical components when evaluating the health impacts of PM_2.5_ exposure.

## 2. Materials and Methods

### 2.1. Inhalation Risk Assessment

The primary exposure pathway for air pollutants such as PM_2.5_ is the respiratory system, with respiratory-related diseases the main health impact [24,25]. Therefore, the extent to which differences in PM_2.5_ concentrations and the chemical compositions of PM_2.5_ affect health in different regions was determined by utilizing health risk assessment techniques proposed by the National Academy of Sciences (NAS) and U.S. Environmental Protection Agency (US EPA) [26,27,28,29]. The US EPA recommends the use of airborne chemical concentrations as exposure indicators for inhalation risk assessment. Consequently, carcinogenic and non-carcinogenic risk assessments were performed based on the concentration of PM_2.5_ chemical components. The human exposure dose was calculated as the average daily dose (ADD) and lifetime average daily dose (LADD), as shown in Equations (1) and (2).

(1) $${ADD}_{i}=\frac{C_{i}\times IR\times ET\times ED\times EF}{BW\times AT}$$

(2) $${LADD}_{i}=\frac{C_{i}\times IR\times ET\times ED\times EF}{BW\times LT}$$

where IR is the inhalation rate (m^3^/day), $C_{i}$ is the pollutant concentration (mg/m^3^), ET is the exposure time (h/d), ED is the exposure duration (years), EF is the exposure frequency (days/year), AT is the average exposure time (h), LT is the lifetime (h), and BW is the body weight (kg).

The exposure factors reported in the equations were set based on a cohort population aged 60 years and older, considering the life expectancy of South Koreans (82.7 years). Uncertainty in the exposure dose estimation was addressed by calculating both the central tendency exposure (CTE) and reasonable maximum exposure (RME), with 50% and 95% considered representative, respectively. However, pollutant concentrations are generally skewed to the left, and using the 50% value may lead to an underestimation of high exposure levels. Thus, the mean was used to indicate pollutant concentrations [30]. Details of the concentrations are summarized in Table S1 (located in the Supplementary Materials) and the exposure factors applied in this study are summarized in Table 1.

The health risks associated with exposure to PM_2.5_ and its chemical components were evaluated using the hazard quotient (HQ) for non-carcinogenic substances and excess cancer risk (ECR) for carcinogenic substances, based on the reference dose (RfD) and cancer slope factor (CSF), respectively. The HQ and ECR were calculated using Equations (3)–(6):

(3) $${HQ}_{i}\;=\;\frac{{ADD}_{i}}{{RfD}_{i}}=\frac{{ADD}_{i}}{{RfC}_{i}\times \frac{IRs}{BWs}}$$

(4) $${ECR}_{i}\;=\;{LADD}_{i}\times {CSF}_{i}={LADD}_{i}\times \frac{{IUR}_{i}\times BWs}{IRs}$$

(5) $$HI=\;\sum_{i=1}^{n}{HQ}_{i}$$

(6) $$TECR=\;\sum_{i=1}^{n}{ECR}_{i}$$

where ${HQ}_{i}$ represents the HQ of pollutant i, ${ADD}_{i}$ is the average daily dose of pollutant i (mg/kg/day), ${RfD}_{i}$ is the RfD of pollutant i (mg/kg/day), ${ECR}_{i}$ represents the excess cancer risk of pollutant i, ${LADD}_{i}$ is the lifetime average daily dose (μg/kg/day) of pollutant i, ${IUR}_{i}$ is the inhalation unit risk (IUR, (μg/m^3^)^−1^) of pollutant i, ${CSF}_{i}$ is the CSF((μg/kg/day)^−1^) of pollutant i, and HI and TECR represent the total hazard index and total excess cancer risk, respectively. HQ and HI values of >1.0 indicate potential concern [35], and the acceptable risk levels for ECR and TECR range from 1.0 × 10^−6^ to 1.0 × 10^−4^ [35].

Inhalation risk assessments were conducted using toxicological data from databases such as the Integrated Risk Information System, Provisional Peer-Reviewed Toxicity Values, Agency for Toxic Substances and Disease Registry, California Environmental Protection Agency, and Health Effects Assessment Summary Table, and the chemical components of PM_2.5_ were identified using the established reference concentration (RfC) and IUR values. Ten substances were selected to indicate the RfC (Al, V, Mn, Ni, Co, As, Mo, Cd, Ba, and Cr) and six for the IUR (As, Cr, Ni, Co, Cd, and Pb) (Table S2, located in the Supplementary Materials). Cr was further evaluated based on its valence state, with Cr(VI) considered the most toxic form [36]. The US EPA recommends using 1/7 of the total Cr concentration to represent Cr(VI) [37], resulting in an estimated Cr(VI):Cr(III) ratio of 1:6 in ambient air [38]. The health risk assessment was extended to include PM_2.5_, emphasizing the need to consider both total PM_2.5_ concentrations and its specific chemical components. As PM_2.5_ is a complex mixture with no established toxicity values, previous studies [21,39] were examined and the South Korean annual standard of 15 μg/m^3^ was adopted as the RfC. Noncarcinogenic risk assessments using HQ were performed to compare health risks across regions in which this standard was exceeded.

### 2.2. Study Design

#### 2.2.1. Sampling Site

**Figure 1:**
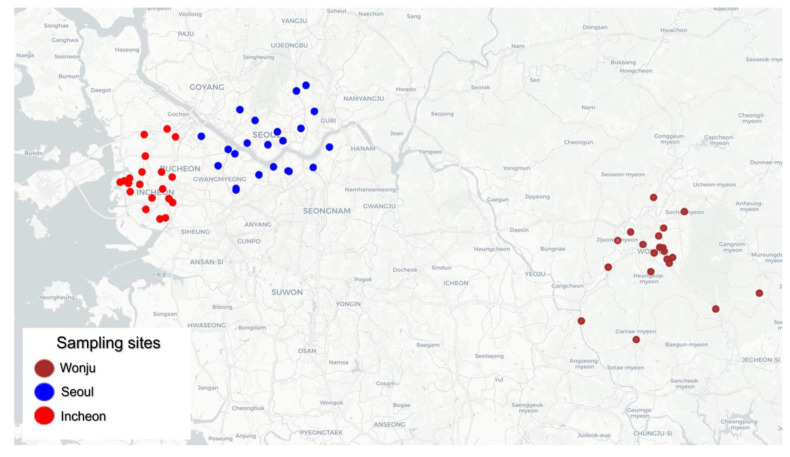
Map of sampling sites.

Three regions were selected to assess the effects of differences in PM_2.5_ concentration and its chemical composition on human health: Seoul, a representative urban area of South Korea; Incheon, an industrial hub adjacent to Seoul with diverse manufacturing activities, including the use of machinery, the metal industry, and electronics development; and Wonju, a region with older industrial complexes that still use older combustion resources, such as charcoal kilns. PM_2.5_ samples were collected from the outdoor air in all three regions from September 2022 to August 2024 (Figure 1), with the outdoor sampling locations selected based on the residences of cohort participants aged 60 and older with degenerative neurological diseases.

##### Seoul

Seoul, the capital of South Korea, had a population of 9.34 million and population density of 15,425 people/km^2^ [40] as of November 2024. The dense population in this area means that it is significantly affected by emissions from various residential and industrial activities. According to the Ministry of Environment, in 2021, Seoul’s PM_2.5_ emissions amounted to 2,605,865 kg, with fugitive dust accounting for 1,220,149 kg (46.8%), making it the largest contributor. Off-road mobile sources, such as construction equipment, contributed 32.5%, whereas on-road mobile sources accounted for 8.9% [41]. Considering that some fugitive dust is traffic-related, it can be inferred that a significant portion of Seoul’s PM_2.5_ emissions are both directly and indirectly linked to transportation, highlighting the need for effective management of air pollution caused by traffic and daily transportation activities.

##### Incheon

Incheon, a port city west of Seoul, had a population of 3.02 million [42] and a population density of approximately 2831 people/km^2^ in November 2024. Since the 1970s, large-scale national industrial complexes such as the Juan, Bupyeong, and Namdong complexes have been established in Incheon and include the production of domestic appliances, metals, automotive industries, and electronics manufacturers. General industrial complexes such as the Seobu and Cheongna industrial zones have also been developed. According to the emissions data, Incheon’s total PM_2.5_ emissions were 2,316,291 kg in 2021, with fugitive dust contributing 40.0%, off-road mobile sources contributing 28.8%, and energy industry combustion contributing 14.2% [41], indicating that the air pollution in Incheon is influenced by traffic, industrial operations, and fossil fuel combustion.

##### Wonju

Wonju, located east of Seoul, is the largest city in Gangwon Province, with a population of 360,000 [42]. It has the lowest population density of the three regions at approximately 412 people/km^2^. Despite its low density, Wonju serves as a key traffic hub that connects the Seoul metropolitan area with Gangwon Province, leading to significant logistics and transportation activities. The region also has older industrial parks and combustion sources such as charcoal kilns are frequently used in the city [43]. In 2019, Wonju’s PM_2.5_ emissions amounted to 471 tons, nearly double that of nearby Chuncheon [44]. The city’s basin topography results in frequent air stagnation events, leading to high PM_2.5_ concentrations [45].

#### 2.2.2. Sampling Method

To accurately assess the regional exposure levels of PM_2.5_, its chemical components, and the health impacts, PM_2.5_ samples were collected outdoors near the residences of cohort participants over the entire study period (September 2022 to August 2024), with sampling conducted at 20 locations per quarter in Seoul (September 2022 to June 2023), Incheon (July 2023 to July 2024), and Wonju (March 2024 to July 2024). Sampling was conducted only on weekdays to account for temporal variation, minimizing bias so that comprehensive results could be obtained.

Sampling was performed continuously for 24 h, starting at 8:00 AM on each sampling day. PM_2.5_ samples were collected using a low-volume air sampler (PMS 204, APM Co., Seoul, Republic of Korea), which is designed based on the US EPA Federal Reference Method for PM_2.5_ and operates at a flow rate of 16.7 L/min (Table S3, located in the Supplementary Materials). Teflon filters (PTFE 2.0 μm, Ø47 mm) were used to analyze any ionic and trace metal components. A total of 200 Teflon filter samples were collected from the three regions over the study period (Seoul, 80; Incheon, 80; and Wonju, 40). To minimize sample loss, the PM_2.5_-laden filters were transported in an icebox with a refrigerant and a temperature of <4 °C was maintained. Antistatic materials and cushioning devices were used to prevent particle loss due to vibration or impact during transportation.

To ensure reliable PM_2.5_ mass concentration measurements, laboratory blanks (LABs) and field blanks (FBs) were prepared for each sampling session, and their weights were used to correct the PM_2.5_ mass concentrations. The LAB filters were stored in the laboratory throughout the sampling period and reweighed after sampling for quality control purposes. The FB filters were transported to the sampling site without exposure and then reweighed to check for contamination during transport. The filter weights were measured before sampling using a robotic weighing system in a controlled weighing room at a temperature of (20 ± 2) °C and relative humidity of (35 ± 5) % following a 24 h conditioning period. The same conditions were used after sample collection to ensure accurate weighing. The PM_2.5_ mass concentration was calculated using Equation (7):

(7) $${PM}_{2.5}(\mu g/m^{3})\;=\;\frac{\left(W_{f}\;-\;W_{i})\;-\;(W_{FBf}\;-\;W_{FBi}\right)}{V_{a}}$$

where $W_{f}$ is the filter weight after sample collection (μg), $W_{i}$ is the filter weight before sample collection (μg), $W_{FBf}$ is the weight of the field blank filter after transport (μg), $W_{FBi}$ is the weight of the field blank filter before transport (μg), and $V_{a}$ is the volume of air sampled (m^3^).

#### 2.2.3. Chemical Analysis and Quality Assurance/Quality Control

An analysis of the chemical composition of the collected PM_2.5_ samples focused on 11 trace elements (Al, V, Mn, Ni, Co, As, Mo, Cd, Ba, Cr(VI), and Pb), which are considered key substances for health risk assessment. The elements were quantified using ED-XRF (Energy Dispersive X-ray Fluorescence Spectrometry, Malvern Panalytical, EPSILON4, Table S4, located in Supplementary Materials) as specified in the guidelines of the Ministry of Environment and National Institute of Environmental Research [46,47]. The concentrations of trace elements were calculated using Equation (8):

(8) $$C\;=\;\frac{(C_{s}-C_{bk})\times A_{u}}{V_{s}}$$

where C is the concentration of trace elements in the air (ng/m^3^), $C_{s}$ is the mass of trace elements collected on the filter (ng/cm^2^), $C_{\;bk}$ is the mass of trace elements on the blank filter (ng/cm^2^), $A_{u}$ is the total filter area used for sampling (cm^2^), and $V_{s}$ is the volume of air sampled (m^3^).

The method detection limit, relative standard deviation, and calibration curve linearity (R^2^) were evaluated for quality assurance/quality control. The relative standard deviation for all trace elements was confirmed to be within 10%, and the method detection limit (MDL) ranged from 0.47 ng/cm^2^ (Cr and Mn) to 59.28 ng/cm^2^ (Al); this is provided in Table S5 (located in the Supplementary Materials). The R^2^ values for the calibration curves were all above 0.99.

#### 2.2.4. Statistical Analysis

An analysis of the regional PM_2.5_ concentration and chemical composition differences was performed using an analysis of variance, which was used to compare the concentration differences in the target substances for health risk assessment, and the Pearson correlation analysis, which was conducted to examine the relationships between PM_2.5_ and its chemical components. Non-detected (N.D.) values obtained in the chemical analysis of the PM_2.5_ samples were excluded from the statistical analyses. By excluding these N.D. values, we aimed to perform a more conservative health risk assessment. Statistical analyses were conducted using SPSS (version 23), with a significance level of 0.05.

## 3. Results and Discussion

### 3.1. Statistical Analysis Distribution of PM_2.5_ and Its Components

The outdoor PM_2.5_ concentrations in the selected regions during the study period are presented in Table 2. The highest concentrations were observed in Seoul (30.11 ± 14.93 µg/m^3^), followed by Incheon (27.17 ± 18.17 µg/m^3^) and Wonju (15.13 ± 11.40 µg/m^3^). analysis of variance confirmed statistically significant differences in the PM_2.5_ concentrations for the three regions (*p* < 0.05). Regarding the chemical composition of PM_2.5_, regional differences were identified for all 11 trace elements (*p* < 0.05), except Ba (*p* > 0.05). These findings highlight the necessity of considering regional variation when assessing outdoor PM_2.5_ exposure. Meanwhile, for Al, the concentration in Wonju was the lowest at 54.10 ng/m^3^. However, since there was only one sample with a concentration above the detection limit, it was considered limited to directly refer to this as the lowest concentration.

The PM_2.5_ concentration in Siheung, located near the study area, was found to be approximately 23.5 µg/m^3^ [48], which is similar to the levels observed in Seoul and Incheon. Meanwhile, when examining the concentrations of heavy metals in PM_2.5_, Pb (1.6 ng/m^3^), Cr (3.0 ng/m^3^), Mn (2.2 ng/m^3^), Ni (4.0 ng/m^3^), and V (1.4 ng/m^3^) were measured. Despite the reduced industrial activity due to COVID-19, V was found to be similar to the other regions in our study. However, the concentrations of other chemical components showed significant differences. The city of Kitakyushu, known for its urbanization and industrial activities, showed PM_2.5_ concentrations ranging from 6.3 µg/m^3^ to 57.5 µg/m^3^, similar to the levels in the three study regions [49]. Furthermore, the median concentrations of heavy metals in PM_2.5_ were found to be Al (41.5 ng/m^3^), Pb (10.5 ng/m^3^), Mn (8.3 ng/m^3^), V (5.7 ng/m^3^), Ni (3.3 ng/m^3^), Cr (3.0 ng/m^3^), Ba (2.1 ng/m^3^), Mo (1.5 ng/m^3^), and As (1.4 ng/m^3^). For Al, Pb, Mn, and As, the concentrations were similar to those found in Wonju, but V was 3–4 times higher than in the three other regions. On the other hand, As concentrations in Seoul and Incheon were approximately 6–7 times higher. These findings reaffirm that while Kitakyushu has mixed characteristics from the study regions, there are differences in the composition of PM_2.5_ depending on the environmental characteristics of each region. Therefore, when understanding the differences in the chemical components of PM_2.5_ between study regions, it is essential to consider the combined effects of regional industrial activities, urbanization, climate, and other environmental factors.

**Figure 2:**
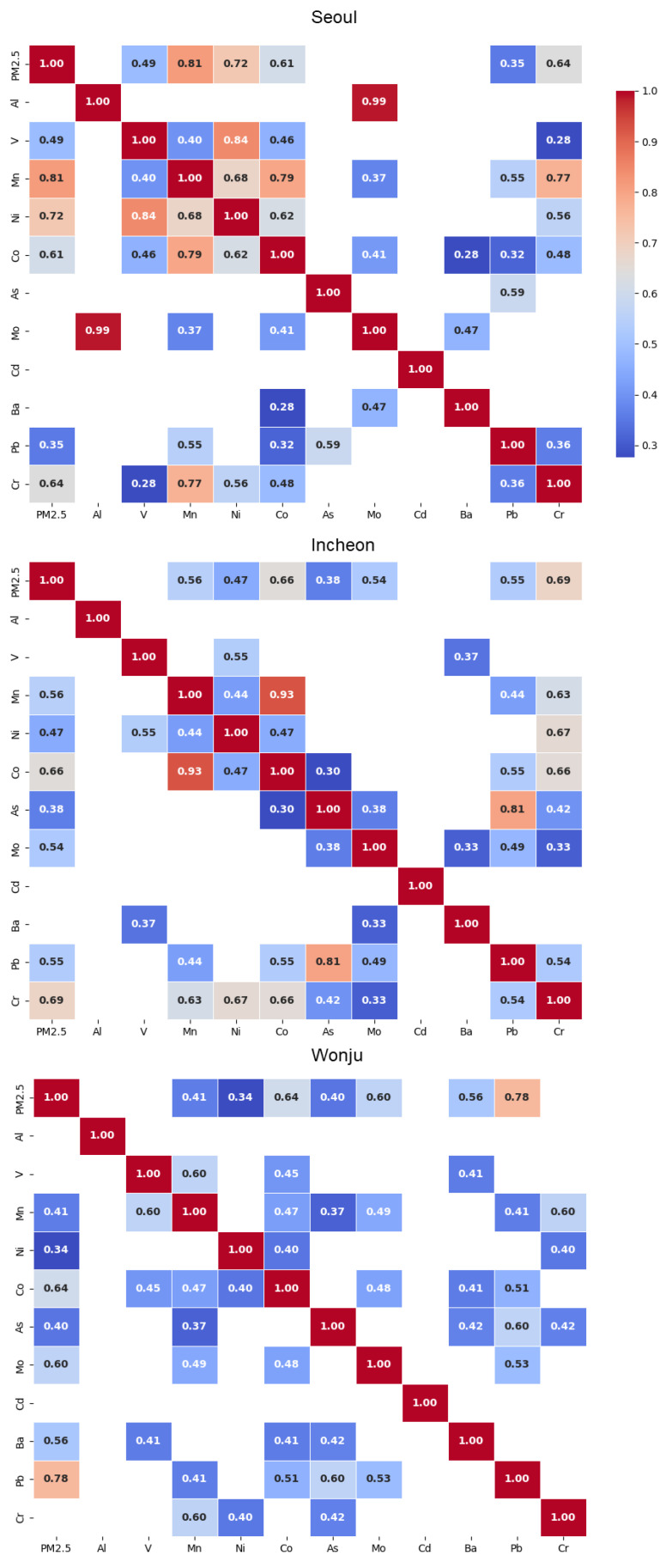
Pearson correlation heatmap of three studied regions.

Correlation analysis between PM_2.5_ and its chemical components in the three regions was used to identify the key factors influencing PM_2.5_ concentrations in each area (Figure 2). In Seoul, Mn showed the highest correlation with PM_2.5_ (r = 0.81), followed by Ni (r = 0.72), Cr (r = 0.64), and Co (r = 0.61). A strong correlation was observed between Ni and V (r = 0.84), which are known to be emitted during fuel combustion (oil combustion) [49,50]. These findings suggest that PM_2.5_ concentrations in Seoul are closely associated with high vehicle traffic and road dust resuspension. Ni, which is typically derived from fuel combustion, is potentially linked to energy consumption in urban areas [49]. Reducing traffic emissions and fuel combustion may therefore effectively mitigate PM_2.5_-related health impacts in this area. Mn, which exhibited the highest correlation with PM_2.5_, has been linked to adverse health effects, such as muscle pain, weakness, emotional disturbances, pulmonary edema, and neurological damage upon long-term exposure [51,52]. Thus, reducing the PM_2.5_ levels could also lead to a decrease in Mn exposure, benefiting older individuals who are at risk of neurological disease. For Al and Mo, a very high correlation coefficient of 0.99 was observed, and these elements are known to potentially originate from common resource sources [53,54]. However, given the limited data for Al in this study, further research is needed to explore the high correlation between these elements.

In Incheon, Cr showed the highest correlation with PM_2.5_ (r = 0.69), followed by Co (r = 0.66), Mn (r = 0.56), Pb (r = 0.55), and Mo (r = 0.54). Mn exhibited a strong correlation with Co, similar to Seoul, suggesting the influence of traffic-related emissions. However, As and Pb also showed a high correlation (r = 0.81) in Incheon, indicating a unique regional emission source. While Pb is associated with coal combustion [55,56], As is known to be emitted from smelting facilities [57]. These results reflect the influence of local industrial activities, particularly coal combustion and smelting processes, on the area’s air quality.

In Wonju, Pb had the strongest correlation with PM_2.5_ (r = 0.78), followed by Co (r = 0.64), Mo (r = 0.60), and Ba (r = 0.56). Previous studies have identified Pb as a tracer of coal combustion [12,56,58], while Ba has been linked to coal-fired power plants and industrial coal combustion [59], as well as brake linings and tire wear in vehicles [60]. These findings suggest that industrial activities and coal combustion contribute significantly to the PM_2.5_ in Wonju. Elevated Pb levels, which are known to cause neurological damage, cardiovascular diseases, and kidney dysfunction, pose a significant health risk to vulnerable populations [61,62]; therefore, the results indicate that the effective management of industrial and traffic emissions is necessary to improve the air quality in Wonju.

### 3.2. Inhalation Risk Assessment

#### 3.2.1. Exposure Assessment

To assess the health impacts of variations in the chemical composition of PM_2.5_ across different regions, the health risk assessment methods recommended by the NAS and US EPA were applied. The assessment included both the carcinogenic and non-carcinogenic risks from PM_2.5_ and its chemical components. Non-carcinogenic risk assessments were conducted for PM_2.5_ and ten elements (Al, V, Mn, Ni, Co, As, Mo, Cd, Ba, and Cr), while carcinogenic risk assessments targeted six elements (As, Cr, Ni, Co, Cd, and Pb).

**Figure 3:**
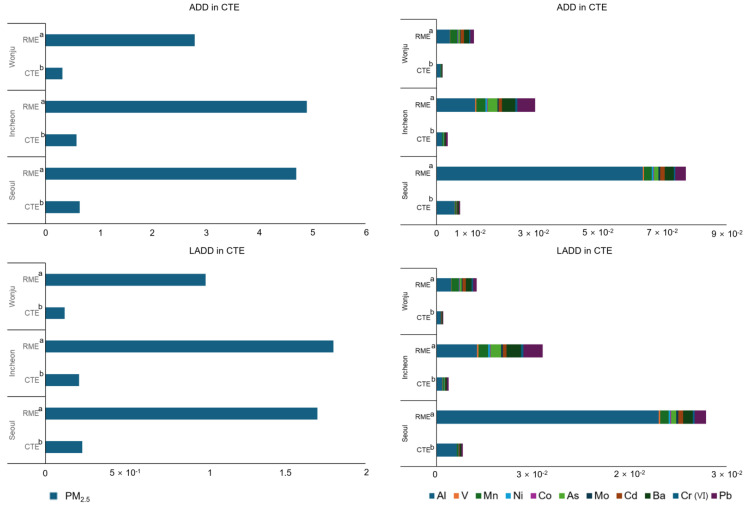
Average daily dose (ADD) and lifetime average daily dose (LADD) across investigated regions by chemical component (µg/kg/day). a = Central tendency exposure, b = Reasonable maximum exposure.

Before calculating the health risks, ADD and LADD were estimated for the target pollutants (Table S6, located in the Supplementary Materials). The PM_2.5_ concentrations and cumulative exposure levels varied significantly across the regions (Figure 3), with the calculated ADD ranked in the order of Seoul (6.4 × 10^−1^ µg/kg/day), Incheon (5.8 × 10^−1^ µg/kg/day), and Wonju (3.2 × 10^−1^ µg/kg/day) under the CTE scenario and Incheon (4.9 µg/kg/day), Seoul (4.7 µg/kg/day), and Wonju (2.8 µg/kg/day) under the RME scenario. When considering the cumulative exposure to hazardous PM_2.5_ components, the CTE scenario followed the same trend as PM_2.5_; however, under the RME scenario, Seoul’s exposure was approximately 2–6 times higher than that in the other regions. These findings suggest that the PM_2.5_ composition differences across the regions significantly impacted the health risk assessments, underscoring the need to consider chemical composition rather than relying solely on PM_2.5_ mass concentration.

#### 3.2.2. Non-Carcinogenic Risk Assessment

An assessment of the non-carcinogenic risks of PM_2.5_ and its chemical components (Table 3) indicated that no component exceeded the HQ threshold (1) in any of the three regions under the CTE scenario. However, under the RME scenario, the HQ values exceeded the threshold in Seoul and Incheon for PM_2.5_ but not for any individual chemical components.

A stricter hazard threshold of HQ = 0.1 [62,63] was investigated. In this case, the PM_2.5_ concentrations exceeded the threshold in Seoul and Incheon under the CTE scenario and all in all three regions under the RME scenario. Five elements (Mn, Ni, Co, As, and Cd) exceeded HQ = 0.1 in Seoul and Incheon, whereas three elements (Mn, As, and Cd) exceeded the threshold in Wonju. These findings suggest that both PM_2.5_ concentrations and chemical compositions should be considered when assessing health risks.

The HI, which represents the sum of the individual HQ values, did not exceed 1 under the CTE scenario in any region. However, under the RME scenario, the HI values exceeded 1 in Seoul (1.3) and Incheon (1.6), whereas Wonju (7.6 × 10^−1^) was relatively high but remained below the threshold. These results suggest that vulnerable individuals may benefit from minimizing outdoor exposure during high PM_2.5_ pollution episodes.

#### 3.2.3. Carcinogenic Risk Assessment

The carcinogenic risk assessment results for the individual chemical components are presented in Table 4. Under the CTE scenario, the estimated ECR values for all the individual substances in Seoul, Incheon, and Wonju were within the acceptable range (1.0 × 10^−5^) or lower, indicating negligible carcinogenic risks. However, under the RME scenario, the ECR for Cr(VI) exceeds the acceptable threshold in all three regions, suggesting a high carcinogenic risk under an RME scenario. Additionally, upon examining the change in the ECR differences between regions in percentage terms, the ECR value for Incheon was approximately 7.0% and 75.6% higher than those of Seoul and Wonju, respectively, which contrasts with the highest PM_2.5_ concentration observed in Seoul based on mass concentration alone. This finding indicates that relying solely on PM_2.5_ mass concentration may underestimate the health risks posed by hazardous chemical components such as Cr(VI), particularly in regions such as Incheon.

These results are similar to previous studies in which the carcinogenic risks of As and Cr were found to be above 1.00 × 10^−6^ [48]. Considering the proximity to the study area, it has been confirmed that As and Cr are the chemical components that require focused management in South Korea. According to previous studies [64], the carcinogenic risk of Cr from industrial and traffic-related sources was found to be more significant than that of other chemical components. Therefore, it is believed that traffic activities have also influenced the results in this study area. Meanwhile, previous research has reported the carcinogenic risks of Cr from PM_2.5_ in Nanjing and Beijing to be 8.70 × 10^−5^ and 2.2 × 10^−5^, respectively [17,65]. In comparison, the carcinogenic risk of Cr in the study area was found to be at a lower level.

The TECR values are 7.5 × 10^−6^, 8.2 × 10^−6^, and 3.8 × 10^−6^ for Seoul, Incheon, and Wonju, respectively, under the CTE scenario, all of which are within an acceptable range. However, the high TECR (2.6 × 10^−4^) obtained for Incheon under the RME scenario is approximately 45.3% higher than that observed in Wonju (1.4 × 10^−4^). These results confirm that the health impact of PM_2.5_ varies depending on the chemical composition distribution of PM_2.5_ across the different regions, expounding the point that the types and concentrations of the chemical components of PM_2.5_ need to be considered in all health assessments. Meanwhile, Incheon has major industrial complexes (in Juan, Bupyeong, and Namdong) from the 1970s [42], making appliances, metals, auto parts, and electronics. In 2021, PM_2.5_ emissions hit 2,316,291 kg, led by fugitive dust (40%), off-road mobile sources (28.8%), and energy combustion (14.2%). These factors—industrial activity, traffic-related pollution, and fossil fuel combustion—likely exacerbate the chemical complexity and concentration of PM_2.5_ in Incheon, contributing to the heightened health risks reflected in the RME scenario. This underscores the necessity of region-specific analyses that account for local pollution sources and demographic factors, particularly in regions such as Incheon, where industrial and urban influences converge to shape air quality and associate health outcomes.

The correlation analysis indicated that Cr, which is associated with high carcinogenic risk, had a strong correlation with PM_2.5_ concentrations in Seoul (r = 0.64) and Incheon (r = 0.69). This suggests that reducing PM_2.5_ concentrations could simultaneously decrease Cr levels, potentially lowering the cancer risk to vulnerable populations.

Certain limitations are associated with this study. First, PM_2.5_ samples were not collected simultaneously across the three regions, limiting the ability to fully account for long-term seasonal and annual variations. Second, PM_2.5_ concentrations were measured near the residences of study participants aged 60 and older, which may not represent the overall regional pollution levels. Despite these limitations, this study confirmed that assessing health risks based solely on PM_2.5_ mass concentrations may underestimate the impacts of specific hazardous components such as Cr(VI) in regions such as Incheon.

## 4. Conclusions

The health risks of PM_2.5_ and its chemical components were assessed for certain areas in Seoul, Incheon, and Wonju to identify regional differences and evaluate the inhalation exposure-related risks. The results confirmed that both the PM_2.5_ concentrations and the chemical compositions varied by region and were influenced by local anthropogenic activities. These regional differences in PM_2.5_ composition are suggested to have varying health impacts. Notably, the carcinogenic risk assessment indicated the highest ECR for Incheon, due to high levels of Cr(VI). However, the results showed the second highest PM_2.5_ concentration for Incheon, underscoring the limitations of relying solely on mass concentration in evaluating health risks. Additionally, the non-carcinogenic risk assessment identified Mn, As, and Cd as key hazardous elements common to all three regions, emphasizing the need for collective management efforts.

This study highlights the necessity of incorporating chemical composition and distribution into health risk assessments rather than relying solely on PM_2.5_ mass concentration. This approach provides accurate insights into regional health risks and contributes to the development of tailored policy measures.

In conclusion, the comprehensive evaluation and management of both PM_2.5_ and its chemical components are essential for enhancing public health and minimizing health risks among vulnerable populations.

## Figures and Tables

**Table 1:** Indoor and outdoor exposure factors.

Exposure Factor	Unit	Value		References
		CTE ^a^	RME ^b^	
IR	m^3^/day	14.10	18.0	[31]
ET	hr/day	2.27	6.26	[31]
EF	days/yr	350	365	[32,33]
ED	yrs	30		[34]
BW	kg	59.78		[31]
AT	h	ED × 365 × 24		This study
LT	h	82.7 × 365 × 24		[31]

^a^ Central tendency exposure. ^b^ Reasonable maximum exposure.

**Table 2:** Average concentration of PM_2.5_ and its chemical components in Seoul, Incheon, and Wonju.

Component	Unit	Concentration						
		N ^a^	Seoul	N ^a^	Incheon	N ^a^	Wonju	*p*-Value
PM_2.5_	µg/m^3^	80	30.11 ± 14.93	80	27.17 ± 18.17	40	15.13 ± 11.40	0.00
Al	ng/m^3^	14	266.59 ± 335.24	5	79.78 ± 55.11	1	54.10	N.A.
V		72	1.67 ± 1.49	71	1.82 ± 1.60	28	0.61 ± 0.34	0.00
Mn		80	17.49 ± 9.35	80	17.34 ± 14.18	39	8.31 ± 8.66	0.00
Ni		79	1.92 ± 1.68	79	2.88 ± 1.99	39	0.87 ± 0.50	0.00
Co		72	1.53 ± 0.81	79	1.42 ± 0.76	37	0.62 ± 0.57	0.00
As		80	7.23 ± 6.43	78	9.07 ± 11.64	36	1.64 ± 2.27	0.00
Mo		35	3.01 ± 2.86	49	2.01 ± 1.85	20	1.54 ± 1.10	0.03
Cd		60	6.61 ± 5.59	60	4.35 ± 4.78	31	4.45 ± 4.04	0.03
Ba		59	18.93 ± 11.40	73	19.38 ± 23.55	27	10.21 ± 7.51	0.06
Cr		80	3.52 ± 1.74	80	4.01 ± 2.25	39	2.44 ± 1.52	0.00
Pb		80	20.55 ± 11.68	79	24.15 ± 23.39	32	6.10 ± 5.12	0.00

^a^ Number of samples.

**Table 3:** Results of the hazardous quotient.

Component	Seoul		Incheon		Wonju	
	CTE ^a^	RME ^b^	CTE ^a^	RME ^b^	CTE ^a^	RME ^b^
PM_2.5_	1.5 × 10^−1^	1.1 × 10^0^	1.4 × 10^−1^	1.1 × 10^0^	7.6 × 10^−2^	6.4 × 10^−1^
Al	4.0 × 10^−3^	4.5 × 10^−2^	1.2 × 10^−3^	8.2 × 10^−3^	8.1 × 10^−4^	3.0 × 10^−3^
V	1.3 × 10^−3^	1.5 × 10^−2^	1.4 × 10^−3^	1.4 × 10^−2^	4.6 × 10^−4^	3.3 × 10^−3^
Mn	2.6 × 10^−2^	1.7 × 10^−1^	2.6 × 10^−2^	1.9 × 10^−1^	1.2 × 10^−2^	1.5 × 10^−1^
Ni	1.0 × 10^−2^	1.1 × 10^−1^	1.5 × 10^−2^	1.2 × 10^−1^	4.7 × 10^−3^	3.0 × 10^−2^
Co	1.9 × 10^−2^	1.4 × 10^−1^	1.8 × 10^−2^	1.2 × 10^−1^	7.7 × 10^−3^	7.8 × 10^−2^
As	3.6 × 10^−2^	3.4 × 10^−1^	4.5 × 10^−2^	7.1 × 10^−1^	8.2 × 10^−3^	1.3 × 10^−2^
Mo	1.1 × 10^−4^	1.0 × 10^−3^	7.5 × 10^−5^	8.1 × 10^−4^	5.8 × 10^−5^	4.3 × 10^−4^
Cd	4.9 × 10^−2^	5.0 × 10^−1^	3.3 × 10^−2^	3.6 × 10^−1^	3.3 × 10^−2^	3.4 × 10^−1^
Ba	2.8 × 10^−3^	2.0 × 10^−2^	2.9 × 10^−3^	2.8 × 10^−2^	1.5 × 10^−3^	1.1 × 10^−2^
Cr(Ⅵ)	3.8 × 10^−4^	1.7 × 10^−2^	4.3 × 10^−4^	2.4 × 10^−2^	2.6 × 10^−4^	1.5 × 10^−2^

^a^ Central tendency exposure. ^b^ Reasonable maximum exposure.

**Table 4:** Results of cancer risk calculation (ECR).

Component	Seoul		Incheon		Wonju	
	CTE ^a^	RME ^b^	CTE ^a^	RME ^b^	CTE ^a^	RME ^b^
As	2.3 × 10^−6^	2.2 × 10^−5^	2.9 × 10^−6^	4.6 × 10^−5^	5.3 × 10^−7^	8.5 × 10^−6^
Cr(VI)	3.2 × 10^−6^	**1.4 × 10^−4,^** ^c^	3.6 × 10^−6^	**2.0 × 10^−4,^** ^c^	2.2 × 10^−6^	**1.2 × 10^−4,^** ^c^
Ni	3.5 × 10^−8^	3.8 × 10^−7^	5.2 × 10^−8^	4.1 × 10^−7^	1.6 × 10^−8^	1.0 × 10^−7^
Co	1.1 × 10^−6^	7.6 × 10^−6^	9.9 × 10^−7^	6.7 × 10^−6^	4.3 × 10^−7^	4.3 × 10^−6^
Cd	8.9 × 10^−7^	8.9 × 10^−6^	5.9 × 10^−7^	6.4 × 10^−6^	6.0 × 10^−7^	6.0 × 10^−6^
Pb	1.8 × 10^−8^	1.3 × 10^−7^	2.2 × 10^−8^	2.3 × 10^−7^	5.5 × 10^−9^	4.4 × 10^−8^

^a^ Central tendency exposure. ^b^ Reasonable maximum exposure. ^c^ Results where ECR exceeded ‘1.00 × 10^−4^’ are highlighted in bold.

## Data Availability

The data presented in this study are available on request from the corresponding author.

## Supplementary Materials

The following supporting information can be downloaded at: https://www.mdpi.com/article/10.3390/toxics13040240/s1, Table S1: Concentration of components used in inhalation risk assessment (µg/m^3^); Table S2: The summary of reference concentration and inhalation unit risk; Table S3: The specification of the measurement device used in this study; Table S4. Energy dispersive X-ray fluorescence analyzer and conditions; Table S5. Quality control results of components analysis for the studied heavy metals; Table S6. The result of average daily dose and lifetime average daily dose (µg/kg/day).

## Abbreviations

The following abbreviations are used in this manuscript:

ADD	Average daily dose
AT	Average exposure time
BW	Body weight
CTE	Central tendency exposure
ECR	Excess cancer risk
ED	Exposure duration
EF	Exposure frequency
ET	Exposure time
FB	Field blank
HI	Hazard index
HQ	Hazard quotient
KOSIS	Korea Statistical Information Service
LADD	Lifetime average daily dose
LAB	Laboratory blank
LT	Lifetime
NAS	National Academy of Sciences
NIER	National Institute of Environmental Research
PM	Particulate matter
PM_2.5_	Particulate matter with an aerodynamic diameter of 2.5 Î¼m or less
RfC	Reference concentration
RfD	Reference dose
RME	Reasonable maximum exposure
TECR	Total excess cancer risk
US EPA	United States Environmental Protection Agency
