# Supplementary material for: Regional Differences in PM2.5 Chemical Composition and Inhalation Risk Assessment: A Case Study of Seoul, Incheon, and Wonju
Source: Toxics. 2025 Mar 24;13(4):240. doi: 10.3390/toxics13040240 (PMC12031520; doi:10.3390/toxics13040240)
Supplement: Supplementary file 1 [file toxics-13-00240-s001.zip › toxics-3487967-supplementary.pdf]

Regional Differences in $PM_{2.5}$ Chemical Composition and Inhalation Risk Assessment: A Case Study of Seoul, Incheon, and Wonju

Seung-Hyun Jung [1], Seon-Ho Baek [2], Shin-Young Park [3], Cheol-Min Lee [3] and Jung-Il Lee [3,*]

1 Climate Technology Center, Korea Testing & Research Institute, Gwacheon 13810, Republic of Korea

2 Air Quality Center, Korea Testing & Research Institute, Gwacheon 13810, Republic of Korea

3 Department of Environmental & Chemical Engineering, Seokyeong University, Seoul 02713, Republic of Korea

Abstract: This study evaluates the chemical components of an aerodynamic diameter less than 2.5 μm (PM2.5) and its health risks in Seoul, Incheon, and Wonju, South Korea. The results revealed significant regional variations, particularly under the reasonable maxi-mum exposure scenario, with Seoul's average daily dose (6.4 × 10 1 μg/kg/day) approximately 2 times higher than Incheon (5.8 × 10 1 μg/kg/day) and Wonju (3.2 × 10 1 μg/kg/day) under the central tendency exposure scenario. Furthermore, exposure to the chemical components comprising PM2.5 can surpass risk thresholds when PM2.5 concen-trations exceed the national standard levels. These findings suggest the potential benefits of preventive measures, such as minimizing outdoor exposure, especially for individuals over 60 years of age, to help reduce health risks. However, further research is needed to confirm the effectiveness of these measures in different regions. The study also highlighted the variation in the health impacts of PM2.5 concentrations and its chemical components across the different regions. The results suggest that relying solely on PM2.5 concentrations for health risk assessments may underestimate the risks associated with carcinogenic components such as chromium (Cr, VI). However, under the reasonable maximum expo-sure (RME) scenario, the excess cancer risk (ECR) for Cr (VI) exceeds the acceptable threshold in all three regions, suggesting a high carcinogenic risk under the RME scenario. For example, the ECR for Cr(VI) in Seoul was calculated as 1.4 × 10 4, Incheon as 2.0 × 10 4, and Wonju as 1.2 × 10 4. Therefore, we emphasize the importance of incorporating both the mass concentration of PM2.5 and its chemical constituents when conducting health risk assessments to inform region-specific health policies to mitigate health risks, particularly for vulnerable populations.

Keywords: PM2.5; region-specific; health risk assessment; Cr (IV); heavy metal

Table S1. Concentration of components used in inhalation risk assessment (㎍/m^3)

Components	Seoul		Incheon		Wonju	
	CTE[a]	RME[b]	CTE	RME	CTE	RME
$PM_{2.5}$	3.0.E+01	6.0.E+01	2.7.E+01	6.2.E+01	1.5.E+01	3.5.E+01
Al	2.7.E-01	8.2.E-01	8.0.E-02	1.5.E-01	5.4.E-02	5.4.E-02
V	1.7.E-03	5.6.E-03	1.8.E-03	5.2.E-03	6.1.E-04	1.2.E-03
Mn	1.7.E-02	3.1.E-02	1.7.E-02	3.5.E-02	8.3.E-03	2.8.E-02
Ni	1.9.E-03	5.8.E-03	2.9.E-03	6.2.E-03	8.7.E-04	1.5.E-03
Co	1.5.E-03	3.0.E-03	1.4.E-03	2.6.E-03	6.2.E-04	1.7.E-03
As	7.2.E-03	1.8.E-02	9.1.E-03	3.9.E-02	1.6.E-03	7.2.E-03
Mo	3.0.E-03	7.4.E-03	2.0.E-03	5.9.E-03	1.5.E-03	3.1.E-03
Cd	6.6.E-03	1.8.E-02	4.4.E-03	1.3.E-02	4.5.E-03	1.2.E-02
Ba	1.9.E-02	3.6.E-02	1.9.E-02	5.2.E-02	1.0.E-02	2.0.E-02
Cr^{6+}	5.0.E-04	8.8E-04	5.7.E-04	1.2.E-03	3.5.E-04	7.7.E-04
Pb	2.1.E-02	4.1.E-02	2.4.E-02	7.1.E-02	6.1.E-03	1.3.E-02

a Central tendency exposure, b Reasonable maximum exposure

Table S2. The summary of reference concentration and inhalation unit risk

Components	RfC (mg/m^3)	Reference[a]	IUR (($\mu g/m^3)^{-1}$)	Reference[a]
$PM_{2.5}$	1.5E-02	KMoE, 2024	N.A.	N.A.
Al	5.0E-03	Ji et al., 2025	N.A.	N.A.
V	1.0E-04	ATSDR	N.A.	N.A.
Mn	5.0E-05	IRIS	N.A.	N.A.
Ni	1.4E-05	ATSDR	2.4E-04	IRIS
Co	6.0E-06	PPRTV	9.3E-03	PPRTV
As	1.5E-05	Cal EPA	4.3E-03	IRIS
Mo	2.0E-03	ATSDR	N.A.	N.A.
Cd	1.0E-05	ATSDR	1.8E-03	IRIS
Ba	5.0E-04	HEAST	N.A.	N.A.
Cr^{6+}	1.0E-04	IRIS	8.4E-02	US EPA RSLs
Pb	N.A.	N.A.	1.2E-05	Ji et al., 2025

a KMoE(Korea Ministry of Environment, Enforcement rule of the clean act conservation act), US EPA RSLs (Regional screening levels, G), IRIS Integrated risk information system, RSL Regional screening levels, ATSDR Agency for toxic substances and disease registry, PPRTV Provisional peer reviewed toxicity values, HEAST Health efects assessment summary table, Cal EPA California environmental protection agency

* Ji, W., Zeng, J., Zhao, K., & Liu, J. (2025). Source apportionment and health-risk assessment of PM2. 5-bound elements in indoor/outdoor residential buildings in Chinese megacities. Building and Environment, 267, 112250.

Table S3. The specification of the measurement device used in this study

PMS-204(APM Co., Ltd, Korea)	
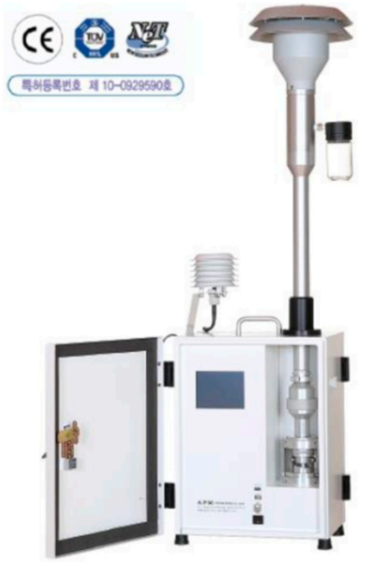 366(W)*465(H)*250(D) MM, 15 kg	
Substance	$PM_{2.5}$
Measurement method	Gravimetrical method
Filter	Teflon(PTFE), Quartz
Operating temperature	-30 ~ 50℃
Internal temperature	± 5℃
Power	110/220 VAC 50-60Hz
Flow rate (Flow control accuracy)	0~20 L/min (±1.5% @ F.S)
Memory	USB Memory 4GB

Table S4. Energy dispersive X-ray fluorescence analyzer and conditions

Energy dispersive X-ray fluorescence analyzer

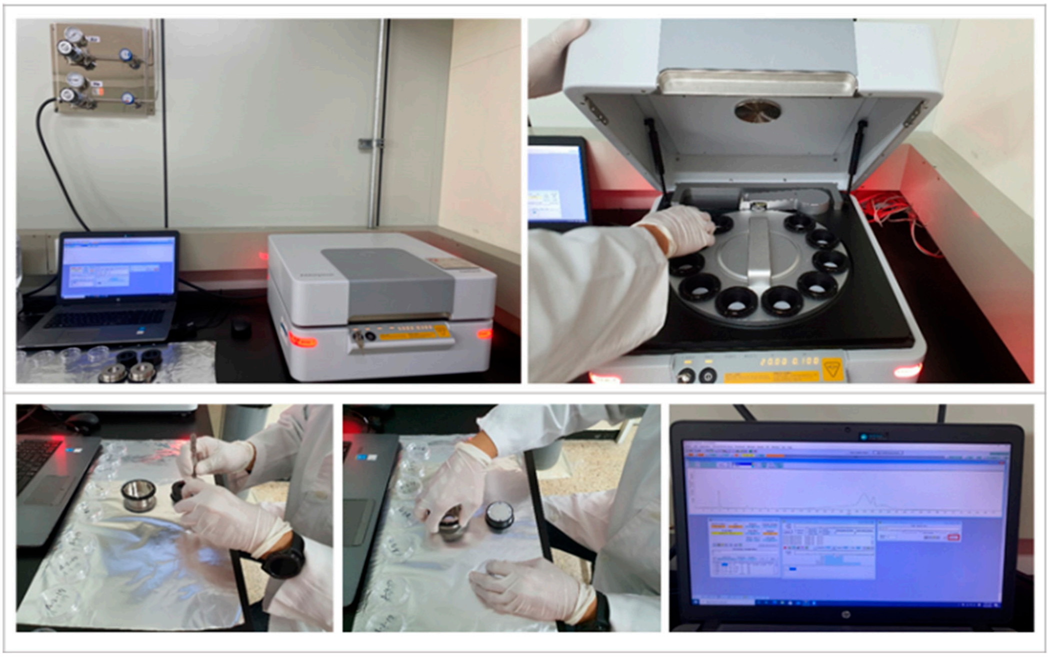

X-Ray Optics	-
Composed of 80° of the X-ray Tube and Detector	Yes
Distance between SDD Detector and Sample	less than 15 mm
Distance between X-Ray Tube Target and Sample	less than 40 mm
X-Ray Tube	-
End-Window Type with Rh Target	End window Ag target, 15 W
50um Be Window, Air Cooled	Yes
X-Ray Generator	-
Range of Voltage	4 ~ 50 kV
Range of Current	0 ~ 3.0 mA
Activated area of 30㎟, 20㎟ Collimated	Yes
Resolution	140eV@Mn Ka 100 kcps
Maximum of Count rate	1.5 Mcps
Window	8 um (0.315 mil) Beryllium

Table S5. Quality control results of components analysis for the studied heavy metals.

Substance	MDL (ng/cm^2)
Al	59.28
V	6.15
Mn	0.47
Ni	1.93
Co	2.47
As	1.43
Mo	3.66
Cd	14.82
Ba	3.86
Cr	0.47
Pb	2.66

Table S6. The result of average daily dose and lifetime average daily dose (μg/kg/day)

Exposure dose	Components	Seoul		Incheon		Wonju	
		CTE[a]	RME[b]	CTE[a]	RME[b]	CTE[a]	RME[b]
ADD	$PM_{2.5}$	6.4.E-01	4.7.E+00	5.8.E-01	4.9.E+00	3.2.E-01	2.8.E+00
	Al	5.7.E-03	6.4.E-02	1.7.E-03	1.2.E-02	1.2.E-03	4.2.E-03
	V	3.6.E-05	4.4.E-04	3.9.E-05	4.1.E-04	1.3.E-05	9.4.E-05
	Mn	3.7.E-04	2.4.E-03	3.7.E-04	2.8.E-03	1.8.E-04	2.2.E-03
	Ni	4.1.E-05	4.5.E-04	6.2.E-05	4.9.E-04	1.9.E-05	1.2.E-04
	Co	3.3.E-05	2.3.E-04	3.0.E-05	2.0.E-04	1.3.E-05	1.3.E-04
	As	1.5.E-04	1.4.E-03	1.9.E-04	3.0.E-03	3.5.E-05	5.6.E-04
	Mo	6.4.E-05	5.8.E-04	4.3.E-05	4.6.E-04	3.3.E-05	2.5.E-04
	Cd	1.4.E-04	1.4.E-03	9.3.E-05	1.0.E-03	9.5.E-05	9.6.E-04
	Ba	4.0.E-04	2.8.E-03	4.1.E-04	4.1.E-03	2.2.E-04	1.6.E-03
	Cr^{6+}	1.1.E-05	4.8.E-04	1.2.E-05	6.9.E-04	7.5.E-06	4.2.E-04
	Pb	4.4.E-04	3.2.E-03	5.2.E-04	5.5.E-03	1.3.E-04	1.1.E-03
LADD	$PM_{2.5}$	2.3.E-01	1.7.E+00	2.1.E-01	1.8.E+00	1.2.E-01	1.0.E+00
	Al	2.1.E-03	2.3.E-02	6.2.E-04	4.2.E-03	4.2.E-04	1.5.E-03
	V	1.3.E-05	1.6.E-04	1.4.E-05	1.5.E-04	4.7.E-06	3.4.E-05
	Mn	1.4.E-04	8.7.E-04	1.3.E-04	1.0.E-03	6.4.E-05	7.9.E-04
	Ni	1.5.E-05	1.6.E-04	2.2.E-05	1.8.E-04	6.8.E-06	4.3.E-05
	Co	1.2.E-05	8.5.E-05	1.1.E-05	7.4.E-05	4.8.E-06	4.8.E-05
	As	5.6.E-05	5.3.E-04	7.0.E-05	1.1.E-03	1.3.E-05	2.0.E-04
	Mo	2.3.E-05	2.1.E-04	1.6.E-05	1.7.E-04	1.2.E-05	8.9.E-05
	Cd	5.1.E-05	5.1.E-04	3.4.E-05	3.7.E-04	3.5.E-05	3.5.E-04
	Ba	1.5.E-04	1.0.E-03	1.5.E-04	1.5.E-03	7.9.E-05	5.7.E-04
	Cr^{6+}	3.9.E-06	1.8.E-04	4.4.E-06	2.5.E-04	2.7.E-06	1.5.E-04
	Pb	1.6.E-04	1.2.E-03	1.9.E-04	2.0.E-03	4.7.E-05	3.8.E-04

a Central tendency exposure, b Reasonable maximum exposure
